# Inhibiting BRAF/EGFR/MEK suppresses cancer stemness and drug resistance of primary colorectal cancer cells

**DOI:** 10.18632/oncotarget.28517

**Published:** 2023-10-04

**Authors:** Astha Lamichhane, Gary D. Luker, Seema Agarwal, Hossein Tavana

**Affiliations:** ^1^Department of Biomedical Engineering, The University of Akron, Akron, OH 44325, USA; ^2^Department of Radiology, Microbiology and Immunology, Biomedical Engineering, University of Michigan, Ann Arbor, MI 48105, USA; ^3^Department of Pathology, Biochemistry, Molecular and Cellular Biology, Georgetown University, Washington, DC 20007, USA

**Keywords:** drug resistance, cancer stem cells, patient-derived tumor model, colorectal cancer, combination treatment

## Abstract

Drug resistance is a major barrier against successful treatments of cancer patients. Gain of stemness under drug pressure is a major mechanism that renders treatments ineffective. Identifying approaches to target cancer stem cells (CSCs) is expected to improve treatment outcomes for patients. To elucidate the role of cancer stemness in resistance of colorectal cancer cells to targeted therapies, we developed spheroid cultures of patient-derived BRAF^mut^ and KRAS^mut^ tumor cells and studied resistance mechanisms to inhibition of MAPK pathway through phenotypic and gene and protein expression analysis. We found that treatments enriched the expression of CSC markers CD166, ALDH1A3, CD133, and LGR5 and activated PI3K/Akt pathway in cancer cells. We examined various combination treatments to block these activities and found that a triple combination against BRAF, EGFR, and MEK significantly reduced stemness and activities of oncogenic signaling pathways. This study demonstrates the feasibility of blocking stemness-mediated drug resistance and tumorigenic activities in colorectal cancer.

## INTRODUCTION

Colorectal cancer is the third most diagnosed cancer and the second leading cause of cancer-related deaths in the world [1]. Aberrant activities of multiple signaling pathways such as epidermal growth factor receptor (EGFR), RAS-RAF, or PTEN-PI3K may promote carcinogenic mechanisms in colorectal tissue [2]. Mutations that constitutively activate RAS, BRAF, or PIK3CA are most common among colorectal cancers with frequencies of 30–50%, 10–15%, and 10–20%, respectively [3]. Two main downstream pathways that harbor mutations in over 50% of colorectal cancers are mitogen-activated protein kinase (MAPK) and phosphatidylinositol 3-kinase (PI3K)/Akt/mTOR pathways [4, 5]. Therapeutic targeting of these pathways was shown to suppress growth of colorectal tumors [6, 7]. Nonetheless, cancer cells often adapt to the treatments and develop resistance through mechanisms such as mutation, amplification, or loss of the target; expression of multidrug efflux pumps; activation of receptor tyrosine kinases; compensatory signaling of alternate pathways; and tumor microenvironment-mediated changes in signaling activities of cancer cells [8]. Drug resistance remains a significant challenge against lasting effects of treatments.

Maintaining monolayer cultures of cancer cells under low-dose or stepwise increases in drug pressure has been widely used to develop a resistant population and study the underlying biological mechanisms. However, this approach primarily relies on cancer cell lines and takes several months to obtain a culture of drug resistant cells. More importantly, monolayer cultures do not mimic the three-dimensional (3D) architecture of tumors in terms of close cell-cell contacts, gradients of nutrients and oxygen, diffusion limitations and non-uniform drug exposure, and cellular heterogeneity. To address this shortcoming, we previously adapted 3D spheroid cultures of colorectal cancer cell lines to long-term cultures under a cyclic treatment regimen that mimics how patients receive chemotherapy [9]. We found that BRAF^mut^ and KRAS^mut^ colorectal cancer spheroids treated with different inhibitors of MAPK pathway showed a strong response during the initial treatment phase but became progressively less sensitive to the drugs during subsequent treatment cycles. Feedback activation of PI3K/Akt, JAK/STAT, and Wnt pathways and gain of a cancer stem cell (CSC) phenotype occurred during the treatment cycles [10–12]. Interestingly, the expression of CSC markers reduced during the recovery cycle but increased during the second round of treatments, suggesting the plasticity of cancer cells in shifting their states and consistent with the proposed role of CSCs in drug resistance and relapse [13–15]. We demonstrated that specific combination treatments suppressed feedback signaling and stemness.

Our previous studies used spheroid cultures of cancer cell lines in cyclic treatments to establish the feasibility of investigating mechanisms of drug resistance and testing the efficacy of different drug combinations [16]. Using primary cancer cells is critical to facilitate translational potential of this approach. However, culturing primary cells is challenging and often not feasible. To overcome this challenge, here we demonstrate the integration of 3D cultures of primary tumor cells, maintained as conditionally-reprogrammed cells (CRCs), into our cyclic treatment regimen. The CRC approach originally used a combination of a ROCK inhibitor and irradiated feeder cells to maintain and expand primary cells in culture [17]. But more recently, a defined growth medium was developed to eliminate the need for feeder cells [18]. We found that primary BRAF^mut^ and KRAS^mut^ colorectal CRC spheroids gain stemness and activate compensatory pathways when treated with MAPK pathway inhibitors. We evaluated different drug pairs against these activities but found that they were ineffective against stemness. Only a clinically used triple drug combination against BRAF, MEK, and EGFR showed significant effects against stemness and signaling pathways. Overall, our approach enables mechanistic studies of drug resistance with 3D cultures of primary cancer cells to develop and test treatments that suppress cancer stemness-mediated tumor cell survival.

## RESULTS

### Characterization of tumor spheroids

**Figure 1:**
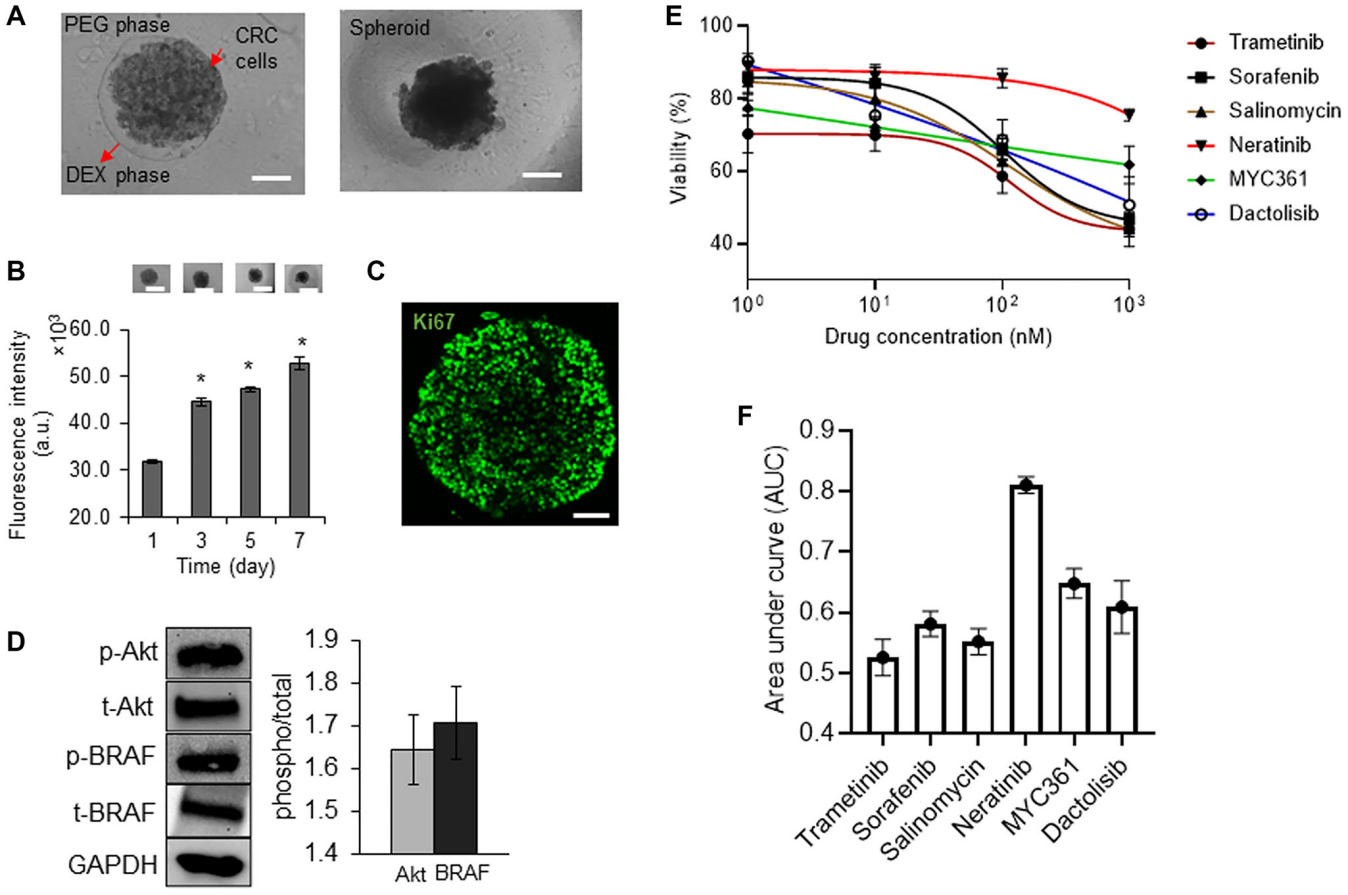
Characterization of tumor spheroids. (**A**) A 0.3 μl drop of the aqueous dextran (DEX) phase containing 1 × 10^4^ CRC2 cells is immersed in the aqueous polyethylene glycol (PEG) phase within a microwell. The drop settles to the well bottom and confines the cells to form a spheroid. Scale bar is 200 μm. (**B**) Temporal metabolic activity of CRC2 spheroids and representative images of spheroids from different days of culture. Error bars represent standard error from the mean (*n* = 8). Scale bar is 300 μm. ^*^Denotes *p* < 0.05 compared to control. (**C**) A CRC2 spheroid immunostained for Ki67 cell proliferation marker on day 3 of culture. Scale bar is 100 μm. (**D**) Representative Western blots for phosphorylated and total levels of Akt, and BRAF in CRC2 cells. Bar graph shows quantified results of activities of the oncoproteins. (**E**, **F**) Dose responses of CRC2 spheroids to different molecular inhibitors and the respective normalized AUC values.

The aqueous two-phase cell patterning confined cancer cells into an aqueous nanodrop immersed in an immiscible aqueous immersion phase to facilitate spheroid formation, while ensuring free diffusive transport of nutrients and waste products between the nanodrop and the immersion phase [19]. After spheroids formed, we maintained them in the complete growth medium (Figure 1A). CRC2 spheroids showed continuous increase of up to ~1.6-fold in cellular metabolic activity over time (Figure 1B). We validated this result by immunostaining the spheroids for a proliferative cell marker, Ki67 (Figure 1C). Next, we evaluated basal level oncogenic signaling in these cells and found significant activities of BRAF and Akt (Figure 1D). To identify effective inhibitors against these pathways in our drug resistance studies, we selected a panel of inhibitors of MAPK, EGFR, BRAF, and PI3K pathways (Supplementary Table 1) and screened them dose-dependently against CRC2 spheroids (Figure 1E). We compared the effectiveness of the inhibitors based on their respective AUC values. AUC ranges from 0 to 1 to indicate complete cell death or viability, respectively, and segregates compounds based on their effectiveness [20]. Trametinib, salinomycin, and sorafenib respectively led to AUC values of 0.52, 0.55, and 0.58 and ranked as the most three effective compounds (Figure 1F). This analysis suggested that suppressing MEK1/2 kinase, which is downstream of both KRAS and BRAF signaling, most effectively inhibits CRC2 proliferation. Thus, we selected MEK inhibition to model drug resistance in subsequent studies.

### Drug resistance and stemness of colorectal cancer cells

**Figure 2:**
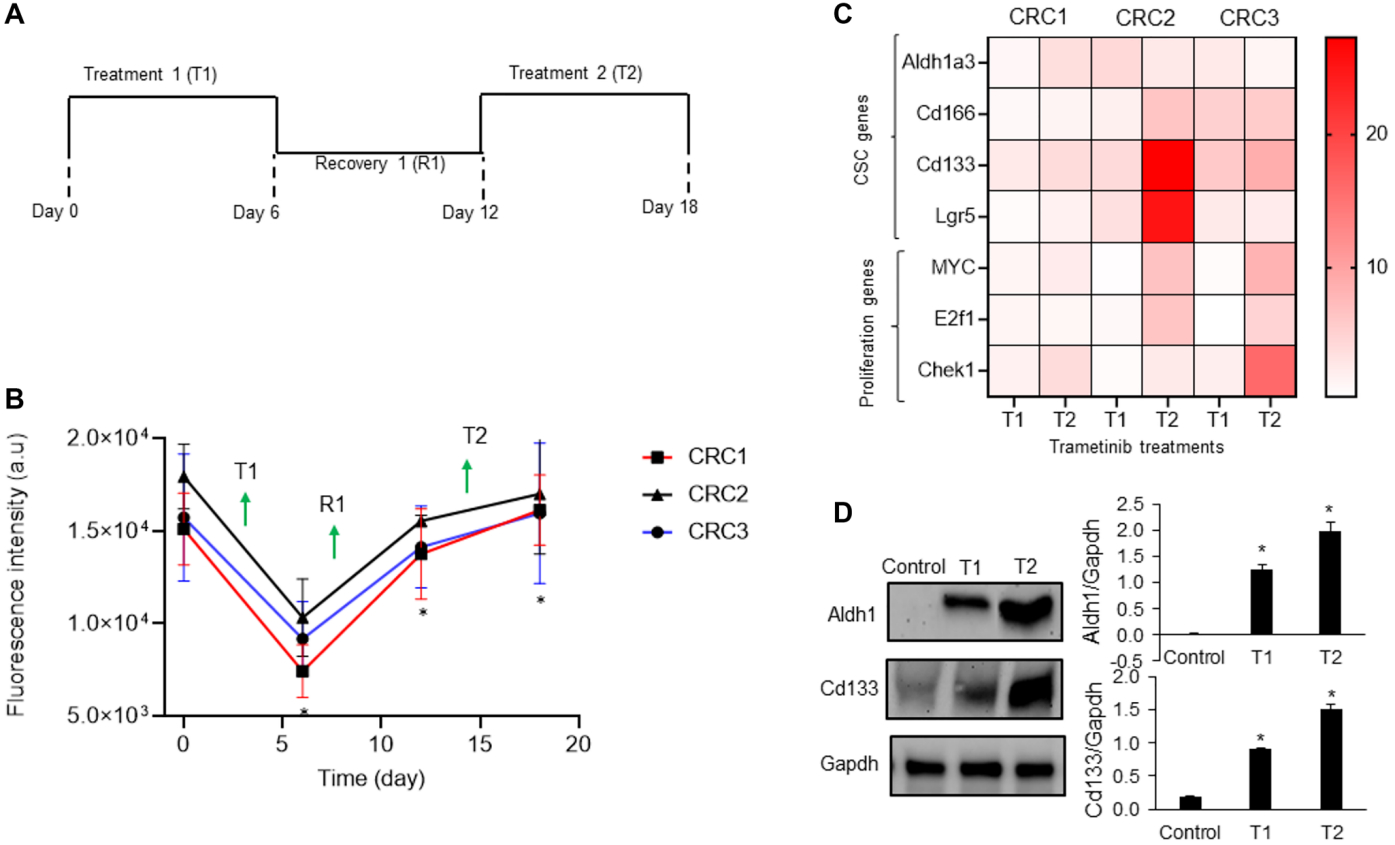
Mechanism of drug resistance of CRC spheroids. (**A**) A cyclic treatment regimen of CRC spheroids consisting of two treatment cycles with 10 nM trametinib separated by a recovery phase. (**B**) Metabolic activities of CRC1, CRC2 and CRC3 spheroids during the cyclic treatment regimen. Error bars represent standard error from the mean (*n* = 8). ^*^Denotes *p* < 0.05 compared to control. (**C**) Heatmap shows the normalized fold change values of prominent CSC gene marker after two rounds of 10 nM trametinib treatment. The fold change values are relative to the control spheroids on day 2. (**D**) Representative western blots for ALDH1, CD133, and Gapdh and the corresponding quantified results of CRC2 spheroids. Error bars represent standard error from the mean (*n* = 2). ^*^Denotes *p* < 0.05 compared to control.

To mimic cyclic chemotherapy, we treated spheroids of all three CRCs with trametinib in the cyclic regimen (Figure 2A) [9]. This approach helped model how cancer cells spared by the treatment develop adaptive resistance. Trametinib potently inhibited cell proliferation during the initial treatment phase (T1) and reduced metabolic activities of spheroids by 2.5-fold, 1.8-fold, and 1.7-fold for CRC1 (KRAS^mut^), CRC2 (BRAF^mut^ and EGFR^mut^), and CRC3 (BRAF^mut^), respectively. However, cells became significantly less responsive to the subsequent treatment (T2) (Figure 2B). At the end of T2, metabolic activities of spheroids respectively increased by 2.6-fold, 1.5-fold, and 1.6-fold for CRC1, CRC2, and CRC3, compared to after T1. This was consistent with our prior studies with cell lines [12], and the lack of response of tumor xenografts to MEK1/2 inhibitors [21].

**Figure 3:**
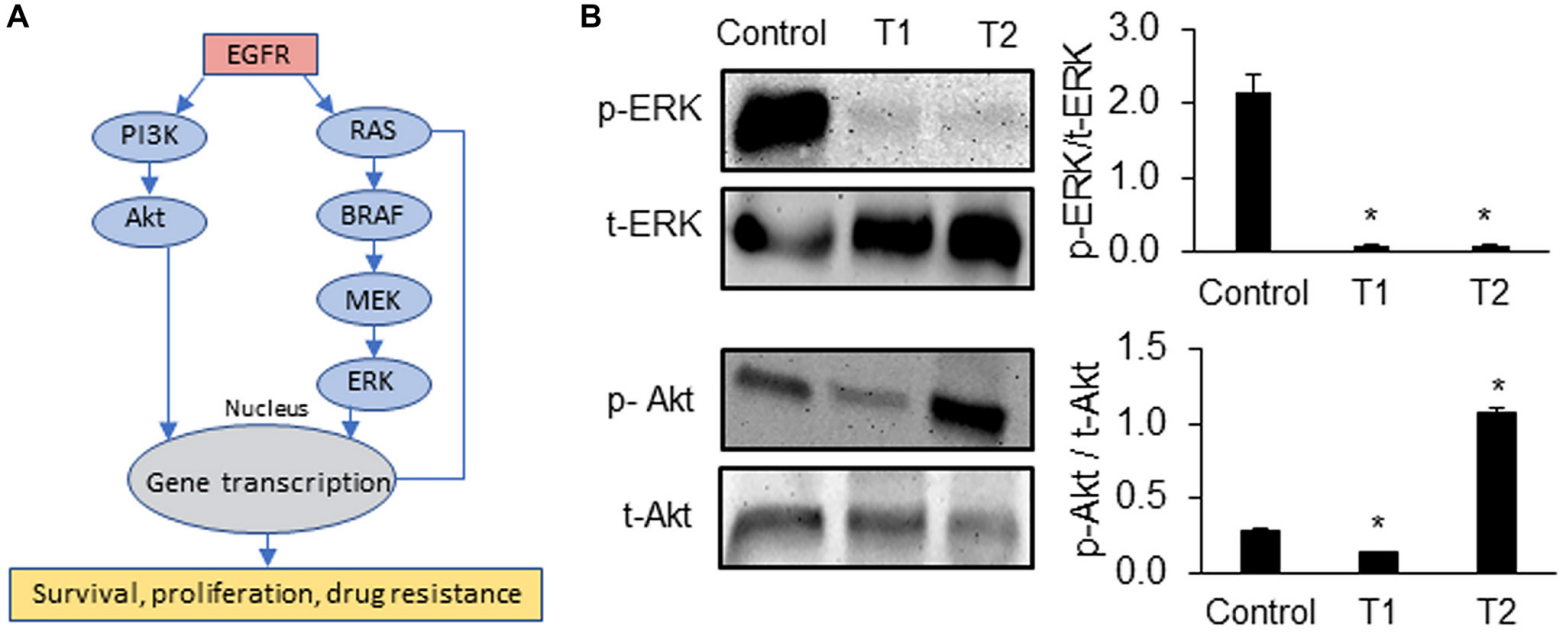
Signaling mechanisms of drug resistant CRCs. (**A**) Oncogenic pathways that promote survival, proliferation, and drug resistance of cancer cells. (**B**) Representative Western blots for ERK and Akt at the end of trametinib treatment 1 (T1) and treatment 2 (T2) of CRC2 spheroids and the corresponding quantified results are shown. Error bars represent standard error from the mean (*n* = 2). ^*^Denotes *p* < 0.05 compared to control.

Motivated by the role of CSCs in chemoresistance [13, 14], we quantified the expression of several prominent CSC gene markers in colorectal cancer, i.e., ALDH1A3, CD166, CD133, and LGR5, following each cycle of treatment [22–24]. In addition, we determined the expression of proliferative gene markers MYC [25], E2F1 [26], and CHEK1 [27]. The expression of these markers following cyclic treatments of spheroids of all three CRCs with trametinib significantly increased (Figure 2C and Supplementary Figure 1). Most of the CSC genes were significantly upregulated in all three CRCs especially after T2. For example, the expression of ALDH1A3 and CD133 increased by 4-fold and the expression of MYC and CHEK1 increased over ~2.5-fold in CRC1, CRC2, and CRC3 after T2 compared to after T1, suggesting gain of stemness and proliferation under treatment pressure. To validate our results, we investigated the protein expression of ALDH1 and CD133 in CRC2 spheroids and found significant increases following T1 and T2 (Figure 2D). Our molecular analysis also showed significantly reduced ERK activity after T1 and T2 (Figure 3). However, PI3K/Akt activity decreased by 2-fold after T1 but increased by 4-fold after T2, consistent with a major resistance mechanism to MEK inhibitors in BRAF^mut^ colorectal cancer cell lines [9, 28]. Overall, these results established that resistance of CRCs to MEK inhibition in cyclic treatments is associated with enrichment of a CSC phenotype and activation of an alternative signaling pathway, PI3K/Akt. Because all three CRCs showed a similar response to MEK inhibition and gain of stemness following the treatments, we selected to only use CRC2 spheroids for our remaining studies.

### Combination treatments to suppress drug resistance of CRCs

**Figure 4:**
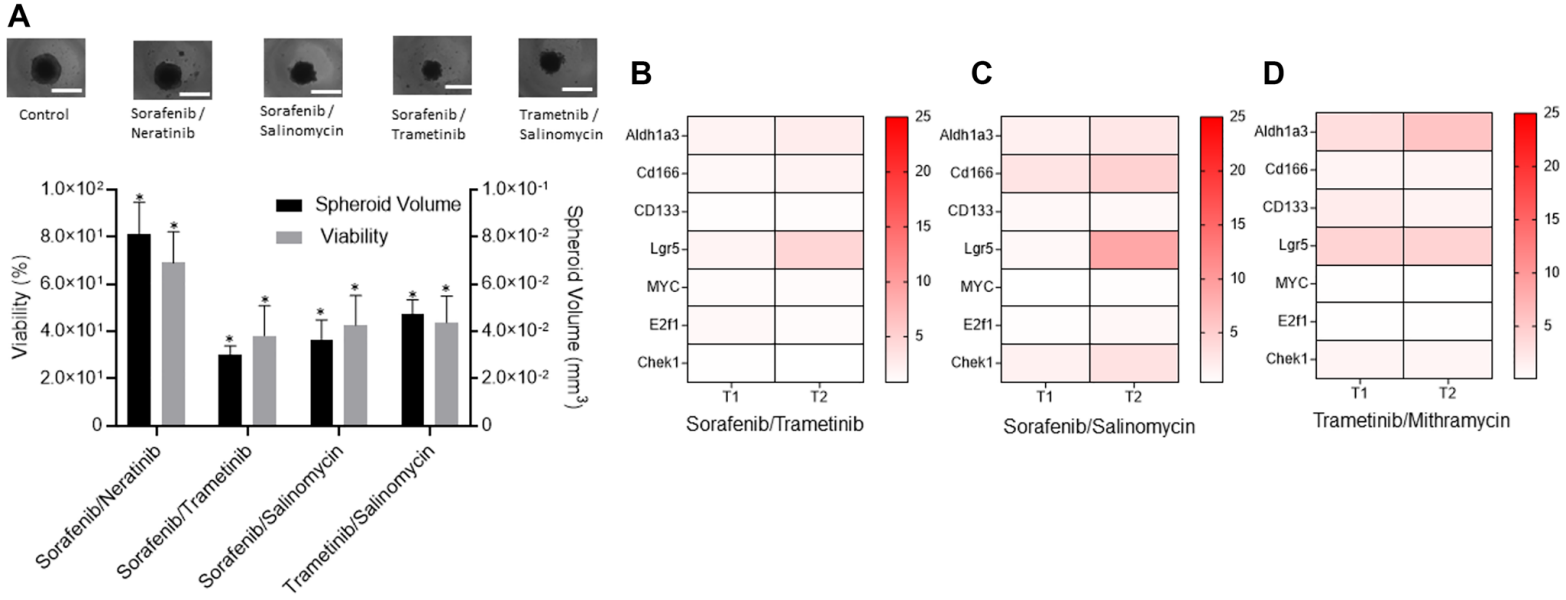
Combination treatments to suppress drug resistance of CRCs. (**A**) Responses of CRC2 spheroids to four different combination treatments measured using percent viability and volume of spheroids with respect to non-treated spheroids. Error bars represent standard error from the mean (*n* = 8). ^*^Denotes *p* < 0.05 compared to control. Images represent control and treated spheroids. (**B**–**D**) The heatmaps show the normalized mRNA fold change values of CSC and proliferation genes in CRC2 spheroids following different combination treatments with (B) 100 nM sorafenib/10 nM trametinib, (C) 100 nM sorafenib/100 nM Salinomycin, and (D) 5 nM trametinib/1 nM mithramycin. The fold change values are relative to the control spheroids from day 2. The colored bar shows the range of fold change values.

Next, we studied whether specific combination treatments could prevent adaptive resistance of CRCs to MEK inhibition. Guided by various clinical trials for colorectal cancer [29–32], we selected and tested combinations of BRAF/EGFR, BRAF/MEK, MEK/WNT, and BRAF/WNT inhibitors at their IC_50_ concentrations against CRC2 spheroids. Combined BRAF/MEK inhibition using sorafenib/trametinib was the most effective and reduced the volume of spheroids by 2.6-fold and cell viability by 1.8-fold (Figure 4A). This was followed by inhibition of BRAF/WNT using sorafenib/salinomycin and MEK/WNT using trametinib/salinomycin that ranked second and third, respectively. We asked to what extent these treatments inhibit stemness of cancer cells. Our analysis of CRC2 spheroids treated with sorafenib/trametinib showed significantly increased expression of ALDH1A3 by 1.9- and 2.5-fold, LGR5 by 1.8- and 4.6-fold, and CD166 by 1.5- and 1.9-fold after T1 and T2, respectively (Figure 4B and Supplementary Figure 2A). The expression of the proliferation gene E2F1 showed a significant increase by 1.5-fold only after T1. Similarly, sorafenib/salinomycin treatment led to a significant increase in the expression of CD166 by 3.0- and 4.7-fold, and ALDH1A3 by 1.8- and 2.7-fold, after T1 and T2, respectively, and LGR5 by 8.8-fold after T2 (Figure 4C and Supplementary Figure 2B). The proliferation gene CHEK1 showed significantly elevated expression by 1.6- and 3.2-fold after T1 and T2, respectively. Trametinib/mithramycin combination decreased the expression of proliferation genes but was ineffective against stemness of cancer cells. Expression levels were significantly elevated for ALDH1A3 by 3.2- and 5.8-fold and for LGR5 by 4.2- and 4.3-fold after T1 and T2, respectively, and for CD133 by 2.0-fold after T1 (Figure 4D and Supplementary Figure 2C).

**Figure 5:**
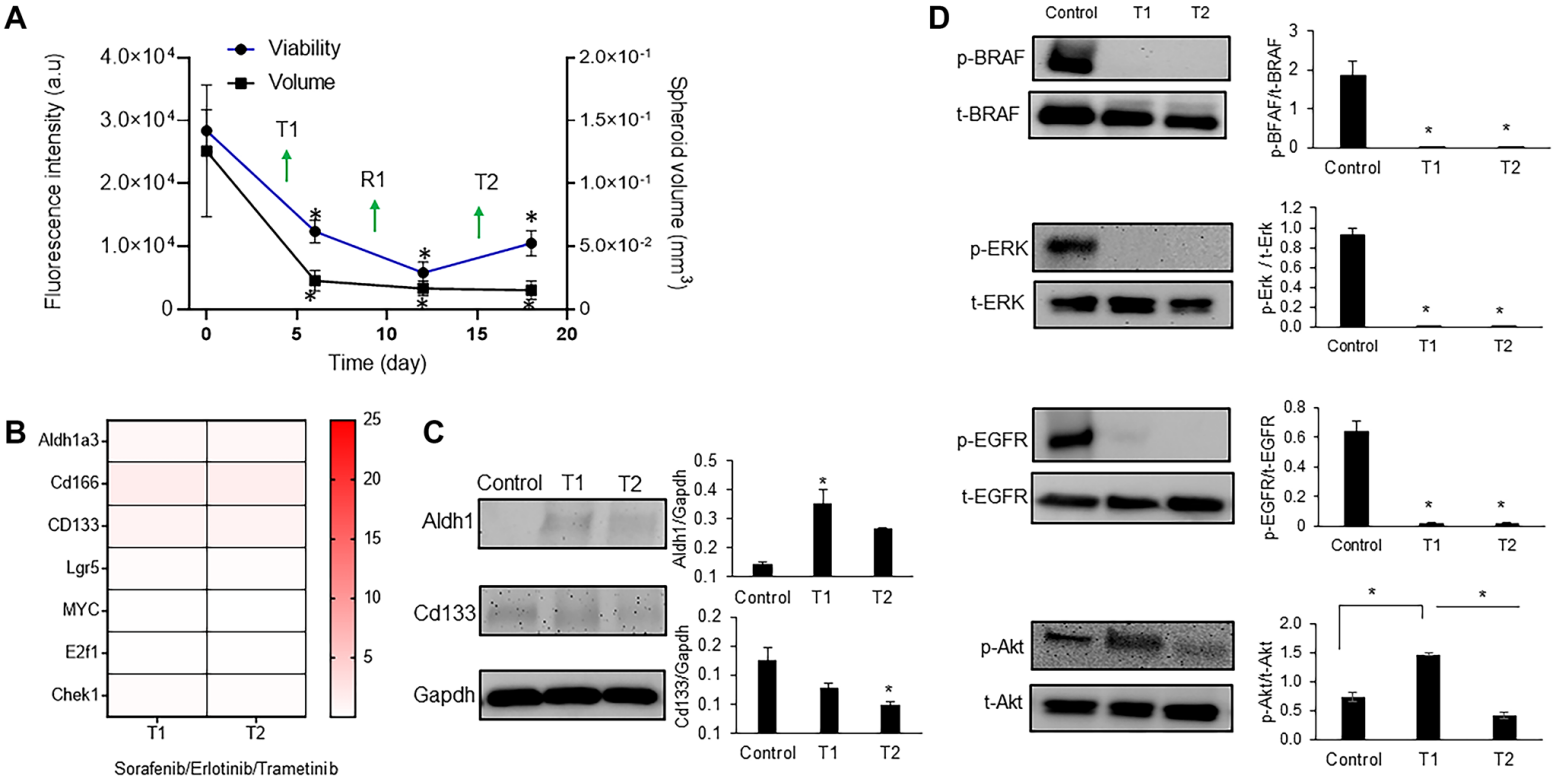
Triple combination to suppress drug resistance of CRC spheroids. (**A**) Metabolic activity and volume of spheroids during cyclic treatment with the triple drug combination. Error bars represent standard error from the mean (*n* = 8). (**B**) Normalized mRNA fold change values of CSC and proliferation gene markers following each round of treatment with 100 nM sorafenib/10 nM trametinib/100 nM erlotinib. The fold change values are relative to the control spheroids. The colored bar shows the range of fold change values. (**C**) Representative Western blots for ALDH1, CD133, and Gapdh and the corresponding quantified results (**D**) Representative Western blots of BRAF, ERK, EGFR, and Akt following cyclic treatments with the triple combination and the corresponding quantified results are shown. Error bars represent standard error from mean (*n* = 2). ^*^Denotes *p* < 0.05 compared to control.

Because these inhibitor pairs did not suppress cancer cell stemness, we aimed to study the effectiveness of a triple drug combination against BRAF, MEK, and EGFR that was used in a clinical trial for patients with BRAF^mut^ colorectal cancer [33]. This regimen was tolerated by patients and led to confirmed and unconfirmed response rates of 21% and 32%, respectively, which are some of the highest response rates with any regimen in BRAF^mut^ colorectal cancer [34]. We treated the BRAF^mut^ CRC2 spheroids with combined sorafenib, trametinib, and erlotinib in the cyclic regimen. The treatment significantly reduced the spheroid volume and metabolic activities of the cells. There was a decrease of 2.2-, 4.8-, and 2.7-fold after T1, R1, and T2, respectively, in the spheroid volume and 5.5-, 7.6-, and 8.3-fold after T1, R1, and T2, respectively, in cellular metabolic activities (Figure 5A). Our results showed a significant decrease in the expression of CSC markers ALDH1A3 by over 2.4- and 3.3-fold, CD166 by over 1.5- and 1.2-fold, and LGR5 by over 4.8- and 10-fold after T1 and T2, respectively, compared to dual combinations (Figure 5B, Supplementary Figures 2, 3). To validate these results at a protein level, we investigated the expression of ALDH1A3 and CD166 markers and found a mild but significant expression of ALDH1 but a significant decrease in expression of CD166 (Figure 5C). The expression of all three proliferative genes also significantly reduced following the triple combination (Figure 5B and Supplementary Figure 3).

Next, we asked to what extent this combination could suppress oncogenic signaling in colorectal cancer cells. Our analysis showed that activities of BRAF, ERK, and EGFR significantly reduced after both T1 and T2 by up to 10-fold (Figure 5D). We have shown in the past that blocking MAPK pathways leads to feedback activity of PI3K/Akt pathway in colorectal cancer cells [9, 12]. This compensatory signaling was active in CRC2 following T1 but was suppressed after T2 by 2.5-fold (Figure 5D). Overall, our results indicate that the triple combination treatment blocked stemness and adaptive responses of BRAF^mut^ colorectal cancer cells.

## DISCUSSION

We demonstrated that monotherapy of three different lines of BRAF^mut^ and KRAS^mut^ patient-derived colorectal cancer cells with a MEK inhibitor led to resistance due to compensatory signaling and gain of stemness, consistent with our previous studies using cell lines [9, 12], and suggesting these mechanisms underlie resistance to MEK inhibition [35, 36]. Elevated expression of CSC markers CD166, ALDH1A3, CD133, and LGR5 was consistent with their role in drug resistance of colorectal carcinoma and correlation with a poor prognosis for patients [31, 37, 38]. Selecting trametinib to model drug resistance was based on its specificity and potency and use in clinical trials of colorectal cancer [39, 40]. Our finding is consistent with studies that showed cyclic treatments of tumor xenografts with MEK inhibitors did not reduce tumor size [21, 41].

Our molecular analysis showed that cancer cells develop adaptive responses to MEK inhibition and gain stem cell-like and proliferative properties. This is consistent with past studies that showed genes such as E2F1 regulate cancer cell proliferation, self-renewal, and drug resistance and act as stemness regulators in tumors [42]. We previously showed MEK inhibition of BRAF^mut^ colorectal tumor spheroids derived from cell lines downregulates ERK activity but activates Akt [9]. This was consistent with our finding with patient-derived cells, suggesting that compensatory PI3K/Akt signaling is a common resistance mechanism in colorectal cancer [43]. Moreover, we showed that dual inhibition of MEK and Akt was unsuccessful in blocking stemness of cancer cells and led to activation of other pathways [12]. Considering that this combination generated excessive toxicity to patients in clinical trials [44], different studies have explored alternative therapeutic options for patients with BRAF^mut^ colorectal cancer. For example, BRAF inhibition alone using vemurafenib demonstrated striking lack of efficacy in colorectal cancer patients, in contrast to BRAF^mut^ melanomas, and produced a response rate of only 5% [45]. This was due to the complex redundant and feedback signaling in colorectal tumors. To evaluate effectiveness of different drug combinations against cancer stemness, we adapted several dual combinations used in clinical trials of colorectal cancers. For example, dual BRAF and MEK inhibition in patients that showed a complete response led to reduced ERK activity relative to pretreatment despite PIK3CA mutations, suggesting that this mutation did not confer resistance to the treatment [30]. However, dual targeting of BRAF and MEK did not suppress CSC gene markers in our study. Motivated by the role of Wnt signaling in stem cells and cancer [46], we evaluated whether dual targeting of BRAF and Wnt could block cancer stemness. However, this approach was also ineffective. Mithramycin A, an FDA approved drug, which was recently shown to inhibit cancer stemness [47], was also ineffective in our study.

Motivated by the results of a phase III BEACON trial that demonstrated a triple drug combination in BRAF^mut^ colorectal cancer significantly increased the response rate and the median overall survival with well-tolerated toxicity [33], we adapted this approach and designed a treatment against BRAF, MEK, and EGFR in our tumor model. Opposing effects of BRAF, MEK, and EGFR inhibitors in normal cells may counterbalance the MAPK pathway effects [34], providing a mechanistic explanation for the observed reduced toxicity in the clinical trial. This strategy generated significant benefits by suppressing expression of ALDH1A3, CD133, LGR5, CD166, and proliferation genes and simultaneously downregulated BRAF, ERK, EGFR, and Akt signaling. Our finding supports the hypothesis that CSCs confer drug resistance and suppressing stemness is a viable approach in BRAF^mut^ colorectal cancer.

In conclusion, this study presented a model of cyclic drug treatment and recovery of patient-derived tumor spheroids and established that single-agent MEK inhibition of colorectal cancer cells lead to adaptive resistance of cancer cells through gain of stemness. A triple combination treatment used in a clinical trial of colorectal cancer patients effectively blocked growth, stemness, and activities of several oncogenic signaling pathways in cancer cells. Our approach to identify mechanisms of drug resistance of patient-derived cancer cells to targeted therapies and develop effective treatments is promising toward cancer precision medicine.

## MATERIALS AND METHODS

### Cell culture and spheroid formation

Three patient-derived conditionally-reprogrammed cells (CRCs) were received from the NCI Patient-Derived Model Repository (PDMR): CN0375 (CRC1), 817829 (CRC2), and 997537 (CRC3). Advanced DMEM/F12 medium was supplemented with 5% fetal bovine serum (FBS) (Hyclone), 0.04% hydrocortisone (Sigma), 0.001% EGF recombinant human protein (Invitrogen), 1% Adenine (Sigma), 1% Pen/Strep (Invitrogen), 1% L-glutamine (Invitrogen), and 0.1% Y-27632 dihydrochloride (Tocris Bioscience) and used to culture the cells. Cells were dissociated with Accutase (Stem Cell Technologies) from 80–90% confluent monolayer cultures in tissue culture flasks. Accutase was neutralized using a stop buffer (90% PBS and 10 % FBS). The cell suspension was centrifuged down at 1000 rpm for 5 min. After removing the supernatant, cells were suspended in 1 mL of the culture medium and counted using a hemocytometer prior to spheroid formation. The culture conditions followed published protocols [17].

A polymeric aqueous two-phase system was used to form tumor spheroids [48, 49]. Bio-ultra polyethylene glycol (PEG) with a molecular weight of 35 kDa (Sigma) and dextran (DEX) with a molecular weight of 500 kDa (Pharmacosmos) were dissolved in the complete growth medium to obtain final stock solutions of 5.0% (w/v) PEG and 12.8% (w/v) DEX. The PEG phase solution was supplemented with 0.24% of methylcellulose powder. A round-bottom ultralow attachment 384-well plate (Corning) was used as a “destination plate”. Each well of this plate was loaded with 30 μl of the aqueous PEG phase medium. A suspension of 1 × 10^8^ cells/ml was prepared, and 100 μl of the suspension was thoroughly mixed with 100 μl of the 12.8% (w/v) aqueous DEX phase medium. This mixing reduced DEX concentration to 6.4% (w/v) and adjusted the density of cells to 5 × 10^7^ cells/ml. A 1% collagen solution (4 mg/ml) was added to DEX phase to facilitate forming spheroids. Each well from one column of a flat bottom 384-well plate (Corning), which was used as a “source plate”, was filled with 25 μl of the resulting cell suspension in the DEX phase. A robotic liquid handler (Bravo SRT) (Agilent Technologies) was used to aspirate 0.3 μl of the suspension containing 1 × 10^4^ cells from each well and dispense it into each well of the destination plate containing the aqueous PEG phase. The destination plate was incubated for 48 hours to allow cells in each well to aggregate into a compact spheroid.

### Metabolic activity and immunohistochemical analysis of spheroids

A total of 8 spheroids were analyzed daily to assess their metabolic activities. Culture medium was renewed every 2 days for 9 days. Metabolic activities of cells in spheroids were determined by adding a PrestoBlue reagent (Life Technologies) to wells at 10% of total volume in each well, incubating the plates for 2 h, and measuring the fluorescence intensity from the wells using a plate reader (Synergy H1M, Biotek Instruments) [50]. Spheroids were harvested on day 3 of culture, fixed with 4% paraformaldehyde, and immunostained for Ki67 (Cell Signaling Technology). Nuclei were stained with Hoechst (Life Technologies). Fluorescence images were captured with a confocal microscope (Nikon A1) at a 10× magnification. FITC filter was used to capture stacks of images with a z-spacing of 20 μm. NIS Elements software was used for image acquisition. Z-projected images were reconstructed by collapsing the stacks in ImageJ (NIH).

### Drug tests, cyclic regimen, and combination treatments

Trametinib, sorafenib, salinomycin, neratinib, MYCi361, dactolisib, vemurafenib, and erlotinib were purchased from Selleckchem. Mithramycin A was purchased from Sigma. All compounds were dissolved in dimethyl sulfoxide (DMSO) except for dactolisib that was dissolved in dimethylformamide. Stock solutions of these compounds were prepared according to the manufacturers’ instructions. Stocks solutions were stored in −80°C. The compounds were tested against tumor spheroids according to our published protocol [11, 51]. For cyclic drug treatments of spheroids, a concentration of 10 nM trametinib was selected from dose-dependent tests. CRC spheroids were subjected to two treatment rounds (T1 and T2), each for 6 days with a treatment renewal after 72 h, and with a recovery phase (R1) for 6 days in between [9]. Prior to the start of the experiments and at the end of each cycle of treatment and recovery, metabolic activities of cells were measured using a PrestoBlue reagent.

For combination treatments, IC_50_ concentrations of the compounds were used: 10 nM trametinib/100 nM salinomycin against MEK/WNT and 140 nM sorafenib/116 nM salinomycin against BRAF/WNT. To evaluate the effect of combined inhibition of MEK and CSCs, a matrix format combination treatment was used, and the fraction of cells affected was computed (Supplementary Figure 4). Based on this analysis and to avoid highly toxic concentrations, a 5 nM trametinib/1 nM mithramycin combination was selected. A triple drug combination with 140 nM sorafenib/10 nM trametinib/300 nM erlotinib was used against BRAF/MEK/EGFR. Combinations treatments were done in the cyclic regimen and viability of cells was measured. The volume of spheroids was determined using their phase images as demonstrated previously [28].

### Quantitative RT-PCR

Gene expression analysis with CRC spheroids was performed after T1, R1, and T2 for each treatment. All fold changes values were expressed relative to that after 24 h, which was used as the control for all three time points. Spheroids were lysed using a Total RNA Kit (TRK) lysis buffer (Omega Biotek) and the lysate was homogenized by passing it through homogenizer mini columns (Omega Biotek). Total RNA was obtained using an RNA isolation kit (Omega Biotek). After removing DNA using RNase-free DNase (Omega Biotek), purity and concentration of isolated RNA were assessed using optical density (OD) 260/280 spectrophotometry (Synergy H1M, Biotek Instruments). cDNA was synthesized from 1 μg of total RNA using random hexamer primers (Roche). Real-time q-PCR was performed in a LightCycler 480 instrument II using a SYBR Green Master Mix (Roche). After combining 50 ng of cDNA with primers and the SYBR Green Master Mix to a final volume of 15 μL, the reactions were incubated at 95°C for 5 min followed by 45 cycles of amplification, that is, at 95°C for 10 s, at 60°C for 10 s, and at 72°C for 10 s. The primer sequences for the genes are listed in Supplementary Table 2. Expression levels of mRNA for different proliferation gene markers were calculated relative to β-actin and hypoxanthine phosphoribosyltransferase (HPRT) using the ΔΔCt method. All the expression values were calculated with respect to control (non-treated) samples. The fold changes in the expression of each mRNA was determined according to the 2^−ΔΔCt^ method [52]. A total of 384 spheroids were collected for each condition and every experiment had two biological and two technical replicates.

### Western blotting

Western blot analysis with spheroids was performed using our established protocol [11]. Primary antibodies were phospho-p44/42 MAPK (pErk 1/2), p44/42 MAPK (Erk1/2), phospho-Akt (Ser473), Akt (pan), phosphor-BRAF (Ser445), BRAF (D9T6S), phosphor-EGFR (Tyr1068), EGFR (Tyr1173), CD133 (D2V8Q), and ALDH1A1 (D9J7R) (Cell Signaling Technology). Solutions of primary antibodies were prepared at concentrations recommended by the manufacturer. Nitrocellulose membranes were incubated overnight at 4°C with primary antibody solutions. After several wash steps, membranes were incubated with a horseradish peroxidase (HRP)-conjugated secondary antibody for 1 h, followed by repeated washing. Detection was carried out using ECL chemiluminescence detection kit (GE Healthcare) with a FluorChem E imaging system (ProteinSimple).

### Statistical analysis

Student’s *t*-test or one-way ANOVA were used to evaluate spheroid volume differences between single-agent treatments and vehicle control, to compare mRNA fold change values between treated and non-treated samples, and to compare protein phosphorylation levels from a drug-treated group to its respective vehicle control group. The analysis was performed in Microsoft Excel. Graphpad Prism 5 was used to fit a four-parameter sigmoidal dose-response curve to metabolic activity data and to determine the area under dose-response curves (AUC).

## SUPPLEMENTARY MATERIALS



## Abbreviations

CSC: Cancer stem cells
EGFR: Epidermal growth factor receptor
MAPK: Mitogen-activated protein kinase
PI3K: Phosphatidylinositol 3-kinase (PI3K)
CRC: Conditionally-reprogrammed cells
3D: Three-dimensional
PEG: Polyethylene glycol
DEX: Dextran
AUC: Area under curve
