# Supplementary material for: Inhibiting BRAF/EGFR/MEK suppresses cancer stemness and drug resistance of primary colorectal cancer cells
Source: Oncotarget. 2023 Oct 4;14:879–89. doi: 10.18632/oncotarget.28517 (PMC10549774; doi:10.18632/oncotarget.28517)
Supplement: Supplementary file 1 [file oncotarget-14-28517-s001.pdf]

Inhibiting BRAF/EGFR/MEK suppresses cancer stemness and drug resistance of primary colorectal cancer cells

SUPPLEMENTARY MATERIALS

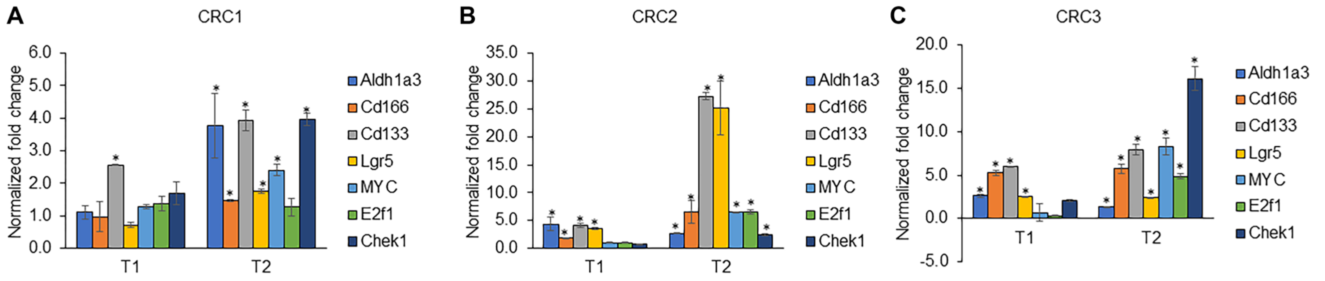

Supplementary Figure 1: mRNA expression of CSC and proliferation genes in spheroids during cyclic treatments with trametinib. The bar graphs show the fold change values (relative to non-treated spheroids) of CSC and proliferation gene markers after treatments with 10 nM trametinib for (**A**) CRC1, (**B**) CRC2, and (**C**) CRC3 spheroids. *Denotes $p < 0.05$ compared to non-treated spheroids. Error bars represent standard error from the mean ($n = 2$, biological replicates).

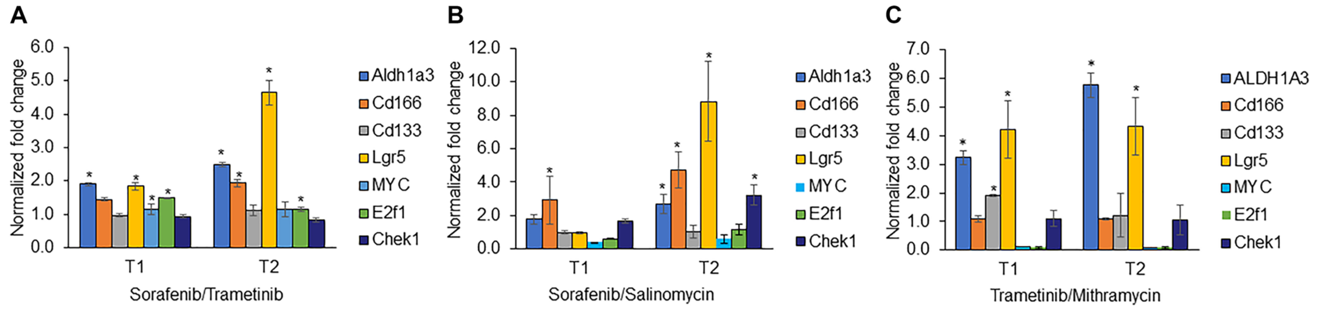

Supplementary Figure 2: mRNA expression of CSC and proliferation genes in spheroids during cyclic treatments with different combinations. The bar graphs show the fold change values (relative to non-treated spheroids) of CSC and proliferation gene markers of CRC2 spheroids after treatments with (**A**) Sorafenib/Trametinib, (**B**) Sorafenib/Salinomycin, and (**C**) Trametinib/Mithramycin. *Denotes $p < 0.05$ compared to non-treated spheroids. Error bars represent standard error from the mean ($n = 2$, biological replicates).

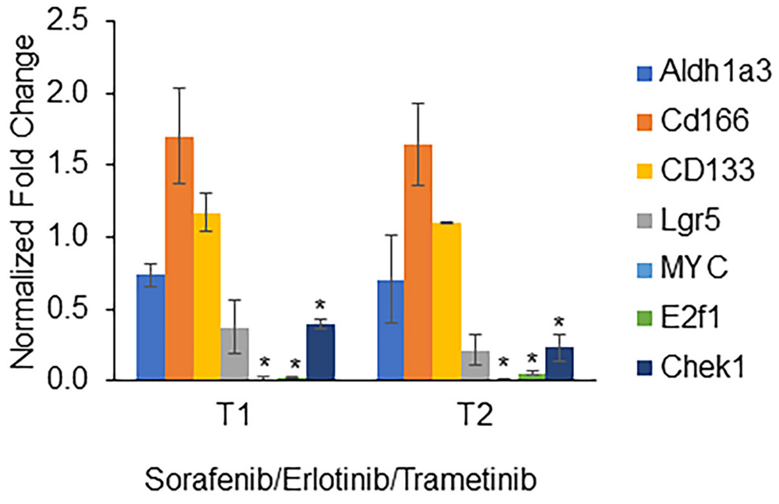

Supplementary Figure 3: mRNA expression of CSC and proliferation genes in spheroids during cyclic treatments with triple drug combination. The bar graph shows the fold change values (relative to non-treated spheroids) of CSC and proliferation gene markers of CRC2 spheroids after treatments with Sorafenib/Erlonitinib/Trametinib. *Denotes $p < 0.05$ compared to non-treated spheroids. Error bars represent standard error from the mean ($n = 2$, biological replicates).

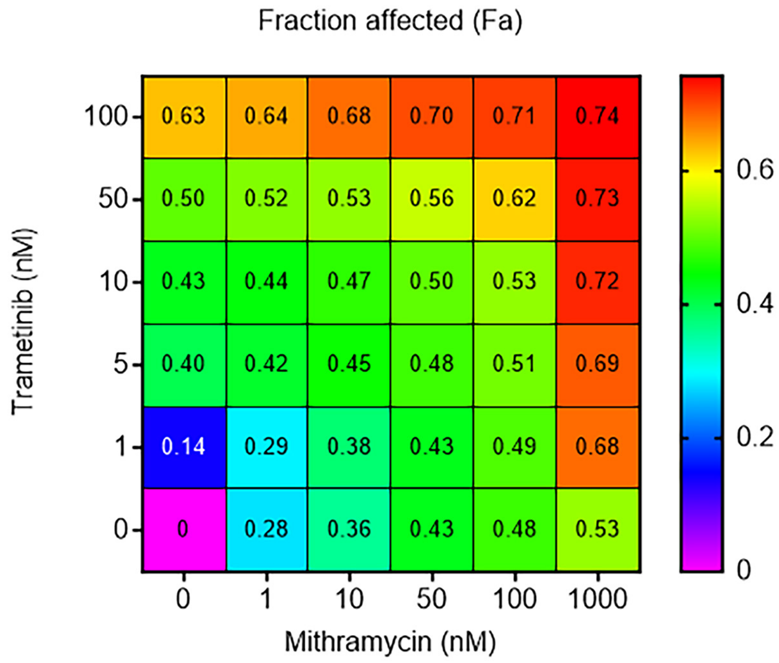

Supplementary Figure 4: Heatmap plot represent fraction of cells affected (Fa) by single-agent and combination treatments (trametinib and mithramycin).

Supplementary Table 1: List of inhibitors and their targets

Compound	Target
Trametinib	MEK1/2
Dactolisib	PI3K/mTOR
Neratinib	EGFR/HER2
Sorafenib	RAF-1, B-RAF, VEGFR-2
Myci361	MYC
Vemurafenib	B-RafV600E
Erlotinib	EGFR
Mithramycin	CSCi
Salinomycin	Wnt

Supplementary Table 2: List and sequences of primers of the CSC and proliferation gene markers

Primer	Sequence	Length
ALDH1A3 F	5′-GGCATACACCTACTCAACTACG-3′	22
ALDH1A3 R	5′-CCTTCTGAGCTAGTATCTTGTCTTTC-3′	26
CD166 F	5′-ATCATACCTTGCCGACTTGAC-3′	21
CD166 R	5′-TCTTTGTAGAGGATCTGAAGGCT -3′	23
BETA ACTIN	5′-ACAGAGCCTCGCCTTTG-3′	17
BETA ACTIN	5′-CCTTGCACATGCCGGAG -3′	17
GAPDH F	5′-ACATCGCTCAGACACCATG -3′	19
GAPDH R	5′-TGTAGTTGAGGTCAATGAAGGG -3′	22
MYC F	5′-TCCTCGGATTCTCTGCTCTC -3′	20
MYC R	5′-TCTTCCTCATCTTCTTGTTCCTC -3′	23
E2F1 F	5′-AAGTCCAAGAACCACATCCAG-3′	21
E2F1 R	5′-CTGCTGCTCGCTCTCCT -3′	17
CHEK1 F	5′-AGTACTGTAGTGGAGGAGAGC -3′	21
CHEK1 R	5′-CCAATACCATGCAGATAAACCAC -3′	23
CD133 F	5′-CCTCTGGTGGGGTATTTCTTT -3′	21
CD133 R	5′-AGGTGCTGTTCATGTTCTCCC -3′	21
LGR5 F	5′-CCAAGGGAGCGTTCACGGGC-3′	20
LGR5 R	5′-CACGTAGCTGATGTGGTTGG -3′	20
